# Artificial intelligence in positive mental health: a narrative review

**DOI:** 10.3389/fdgth.2024.1280235

**Published:** 2024-03-18

**Authors:** Anoushka Thakkar, Ankita Gupta, Avinash De Sousa

**Affiliations:** ^1^ MINDLab, The MINDS Foundation, Mumbai, India; ^2^ Desousa Foundation, Mumbai, India

**Keywords:** artificial intelligence, positive mental health, ethics, machine learning, mental health

## Abstract

The paper reviews the entire spectrum of Artificial Intelligence (AI) in mental health and its positive role in mental health. AI has a huge number of promises to offer mental health care and this paper looks at multiple facets of the same. The paper first defines AI and its scope in the area of mental health. It then looks at various facets of AI like machine learning, supervised machine learning and unsupervised machine learning and other facets of AI. The role of AI in various psychiatric disorders like neurodegenerative disorders, intellectual disability and seizures are discussed along with the role of AI in awareness, diagnosis and intervention in mental health disorders. The role of AI in positive emotional regulation and its impact in schizophrenia, autism spectrum disorders and mood disorders is also highlighted. The article also discusses the limitations of AI based approaches and the need for AI based approaches in mental health to be culturally aware, with structured flexible algorithms and an awareness of biases that can arise in AI. The ethical issues that may arise with the use of AI in mental health are also visited.

## Introduction

“What is AI?” This has been a popular question since the inception of AI or Artificial Intelligence as a discipline in the 1950s (1). There are many proposed definitions of AI, however, most of them are aligned around the concept of machines capable of human behaviors or creating computer programs (2). The founding father of this discipline, John McCarthy (3) described this process as “that of making a machine behave in ways that would be called intelligent if a human were so behaving.” The goal of AI, according to him, was to develop machines that behave as though they were intelligent.

Over the last couple decades, AI has evolved-from playing with toy problems like chess, to acquiring new skills and seeking to discover its own limits. After 60 years, AI has made its way into industries and the consciousness of people (4). It is only now in the 21st century that this discipline has transformed every aspect of our life in such a significant way, that it is referred to as the “*Age of AI*” (5).

Unlike computer science, AI has evolved with a more fluid definition, owing to the different conceptions of intelligence. It was conceived as a computer system that is similar to the human mind in numerous ways (6). AI is an umbrella term that encompasses a wide range of approaches and techniques to develop computational systems which perform cognitive processes and tasks that are characteristic to humans. Examples of such processes and tasks include learning, reasoning, problem solving, drawing inferences and generalization (2).

### Background, history and evolution of AI

The history of AI dates back to the 1950s, a time marked by the initial development of machines with the acumen for human-like decision making and reasoning. This period witnessed significant landmarks, notably the introduction of Unimate in 1961, an industrial robot arm, as well as the creation of Eliza in 1964, a communication-based chatterbot. These headways worked towards laying the bedrock for future strides in robotics and AI. However, in spite of progress in the engineering field during this time, the medical field was slow to adopt AI (7).

Modern concept of AI emerged in the mid-20th century, when Alan Turing, a scholar in this field laid down the theoretical groundwork for AI. This was achieved through his pioneering work on the “Turing Test” and his concepts of universal computing machines (8). The term AI was first coined by John McCarthy in 1956 as “the science and engineering of making intelligent machines,” marking the establishment of the field as a distinct discipline (9).

The progress of AI can be characterized by a number of critical milestones. The Darthmouth Workshop in 1956, which is often considered to be the birthplace of AI, brought together like-minded researchers to explore the possibilities of developing machines mimicking human intelligence. The next couple of decades witnessed some more breakthroughs in this area of research, with systems to manipulate symbols and follow logical rules being designed (10).

In the 1980s, we saw a rise in expert systems, which aimed at capturing human expertise in specific domains using rule-based systems (11). Limitations in dealing with the uncertainty and complexity of real-world situations however, lead to the AI winter. This was a period of dampened enthusiasm and reduced funding in the field of AI which lasted from the 1980s to early 1990s (12).

The resurgence of AI in the late 20th century can be attributed to the advancements in machine learning, like the ability of neural networks to model complex patterns. With the field of machine learning gaining momentum and the development of algorithms, the concept of neural networks showed a rise in the 1990s (13).

The 21st century witnessed a crucial breakthrough in AI as a result of the availability of improved computational resources and massive datasets. Deep learning techniques, like language processing and image recognition revolutionized fields like language understanding and computer vision (14). Seminal advancements in AI including IBM's Watson, an open-domain question-answering system that won a game show in 2011, allowed for the rapid growth of AI in medicine as well as other industries.

## Use of AI in industries

AI has grown exponentially as a transformative technology and is increasingly present in our daily lives-across different industries (15). AI is changing traditional processes, enhancing decision making capabilities and unlocking newer avenues for novelty and innovation. As AI continues to evolve and reshape the way business is conducted, wecan expect a larger shift from industries to a landscape with intelligent systems that are driven by machine learning techniques and advanced algorithms.

The integration of AI into industries consists of a wide spectrum, from healthcare to recruitment, logistics, education, transportation security, e-government, and public sectors. Current advances in the development and applications of AI systems, such as machine learning, deep learning, autonomous or semi-autonomous systems, reshape science and society, creating new opportunities in the way we live, work, travel and do business.

Financial institutions have been employing AI algorithms to detect any fraudulent activities, risk assessment and mitigation, and for enhancing security and stability within the sector (16). In healthcare, AI-powered diagnostic tools are leveraging image recognition and natural language processing to assist medical professionals in accurate disease detection and treatment planning (17). The manufacturing industry has witnessed the implementation of AI-driven maintenance, which in turn has led to a reduction in the downtime and optimizing production processes (18). Transportation and logistics are benefiting from AI-powered route optimization and autonomous vehicles which promise a safer and more efficient mobility solution (19).

The introduction of AI into industries also empowers decision-makers with a more informed decision based on data-driven insights. This in turn enables more informed and strategic choices. Machine learning algorithms for example can process vast volumes of data to uncover patterns and trends, facilitating predictive and prescriptive analytics that drive operational efficiency (20). Through its functioning, AI contributes to resource optimization and cost reduction, ultimately bolstering competitiveness.

A popular form of AI includes machine learning, which learns from imputed data or from the machines experiences and is then taught values via conditioning (21). This can be used in various capacities like voice recognition software (example Alexa and Siri), to medical diagnosis through pattern recognition techniques (22). Other examples of AI in various industries includes AI rapper FN Meka who has signed to Capitol Records and research at Columbia University that resulted in Teaching robots to visual themselves through AI (23). In a 2022 survey consisting of 850 organizations and 18 geographies, 77% reported prioritizing AI regulations as company-wide policies and 80% stated they would invest in the development of ethical AI (24).

Just like most other fields, mental health care too has been impacted by the revolution in digital technology, specifically AI. The emergence of digital mental health as a sub-field signifies the integration of technology into mental health care practices, and within this domain, AI-driven solutions have become an important contributor to positive mental health outcomes (25). The mental health landscape has witnessed a paradigm shift over the last couple years, with technological advancements playing an important role in enhancing accessibility, personalization, and efficacy of interventions. The application of AI in positive mental health is multifaceted, ranging from the development of applications and websites that make use of chatbots and virtual assistants to offer immediate mental health support, someone to talk to in times of distress and identifying potential mental health issues. Machine learning algorithms are also being used to tailor personalized therapeutic interventions, while monitoring real-time data through wearable devices for better informed decision-making. In the context of positive mental health, AI-driven interventions extend beyond clinical settings to wellness applications, delivering tailored recommendations and mindfulness exercises. The purpose of this review is to focus on the ways AI is being used in positive mental health.

## Understanding artificial intelligence and its application in mental health care

### Artificial intelligence and its key components

Artificial Intelligence (AI) refers to the field of computer science and technology that aims to create systems and machines capable of performing tasks that typically require human intelligence, such as understanding natural language, recognizing patterns, making decisions, learning from experience, and adapting to new situations, all achieved through a combination of algorithms, data processing, and iterative improvement processes (9). Following are the notable key components of AI.

Machine Learning (ML) is a subset of AI that involves the development of algorithms that allow systems to learn from data and improve their performance over time without being explicitly programmed (26). It encompasses various techniques like supervised learning, unsupervised learning, deep learning, and reinforcement learning. ML algorithms can analyze patient data, such as electronic health records and behavioral patterns, to assist in diagnosing mental health conditions like depression, anxiety, and schizophrenia (26). They can also predict the risk of developing certain disorders based on historical data.

Additionally, they can help tailor treatment plans by analyzing patient data to recommend specific therapies, medications, or interventions based on individual characteristics and response patterns (25).

In Supervised Machine Learning (SML), the data are pre-labeled beforehand, for example, distinguishing between the diagnosis of major depressive disorder (MDD) and the absence of depression. The algorithm acquires the capacity to establish the most accurate between input features extracted from various sources of data, including sociodemographic, biological, and clinical measures (27). This intricate linkage of features facilitates the algorithm's adeptness in generating highly accurate predictions within these designated categories (28). It is important to note that the labels can be categorical (i.e., MDD or not or continuous (i.e., along the spectrum of severity) in nature. The machine undergoes supervised ML because the labeled data serves as guidance (“teacher”) for the algorithm, teaching it how to assign labels to the data, enabling the algorithm to learn how to link features with specific labels; subsequently, having learned from extensive labeled training data, the algorithm is assessed using unlabeled test data to ascertain its ability to accurately classify the desired outcome, such as MDD (25).

Conversely, in Unsupervised Machine Learning (UML), the algorithms function without predefined labels, enabling them to detect similarities among input features and unveil the inherent data patterns. However, they lack the ability to associate features with a known label (29). The absence of labels presents a greater challenge for UML; however, it offers the advantage of uncovering the inherent structure within a dataset without significant pre existing biases. For instance, extensive feature datasets, such as neuroimaging biomarkers, possess the potential to unveil insights about unknown subtypes within psychiatric disorders like schizophrenia. UML can aid in identifying groups of biomarkers that define these variations, thereby contributing to prognosis and optimal treatment strategies (25).

Artificial Neural Networks (ANNs) and Deep Learning (DL) are based on the structure and functioning of the human brain, DL involves handling complex and raw data, without human guidance, by employing ANNs that resemble the manner in which a human brain thinks (30). This enables them to model complex patterns and relationships in data processed through multiple “hidden” layers (30–32). Deep learning techniques can be used for image analysis in brain scans (MRI, CT, etc.), aiding in the identification of structural abnormalities linked to mental health disorders.

Natural Language Processing (NLP) is another subfield of AI that enables machines to process, comprehend, interpret, and generate human language. Researchers refine and make use of such tools in real-world applications, creating spoken dialogue systems and speech-to-speech translation engines, mining social media for information about health or finance, and identifying sentiment and emotion toward products and services (33). NLP can significantly aid in analyzing written or spoken language to detect emotional states and changes, assisting clinicians monitor patients' mental well-being through texts, chats, or speech. Furthermore, the integration of AI-powered chatbots amplifies this capacity by engaging users in text-based dialogues, thereby delivering real-time assistance, coping mechanisms, and even recommendations for consultation with mental health professionals.

Reinforcement Learning (RL) is a type of ML algorithm that involves training an agent to make sequential decisions by interacting with an environment. The algorithm acts as an agent in an interactive environment that learns by trial and error using rewards from its own actions and experiences (25). RL offers a dynamic framework for tailoring therapeutic interventions. For instance, AI-driven applications, such as virtual reality exposure therapy, can be designed to adjust exposure levels in response to patient reactions. This ensures that the intensity of exposure is optimized to minimize distress while still facilitating progress (34). The AI agent learns from patient feedback, continually refining the intervention strategy over time.

Computer Vision is an AI subfield that encompasses the development of computational systems that facilitate the interpretation and comprehension of visual data from the environment, similar to how humans perceive images and videos. Its practical implementations include image recognition, object detection, facial recognition, and autonomous vehicular systems. Computer vision can be useful in analyzing facial expressions and gestures to infer emotional states, aiding in the assessment of patients' emotional well-being. Additionally, computational intelligence has found application in the detection and assistance of autism spectrum disorder (ASD) (35).

In the field of mental health care, these elements collaborate in many innovative ways. From personalized interventions to predictive analytics, the integration of AI technologies offers promising avenues to strengthen positive mental health outcomes. Further exploration into the landscape of AI in mental health care reveals how these technologies are reshaping the way we approach and support mental well-being (25).

## Overview of AI applications in mental health care

• Awareness: How AI can help in increasing awareness about mental health issues? Through the utilization of advanced technologies such as natural language processing, sentiment analysis, and data mining, AI-driven initiatives have demonstrated remarkable capabilities in disseminating accurate information, combating stigma, and promoting dialogue surrounding mental health. This intersection of AI and mental health awareness presents an innovative avenue with the potential to revolutionize how society perceives and discusses mental health issues. The ability of AI to process and analyze vast amounts of data, coupled with its capacity to interact with individuals, offers promising prospects for creating impactful awareness campaigns. A noteworthy application of AI in mental health awareness is the analysis and regulation of social media data. Platforms like Twitter and Instagram provide a wealth of user-generated content that can be harnessed to gauge public sentiment toward mental health topics. AI-powered sentiment analysis algorithms can discern emotions expressed in posts, enabling researchers and mental health organizations to monitor shifts in public discourse and pinpoint key topics that require targeted awareness initiatives (36). This analysis contributes to a better understanding of prevailing attitudes, misconceptions, and evolving perceptions related to mental health. AI-driven chatbots have also emerged as interactive tools to disseminate accurate information and educational resources on mental health. Such AI-driven agents engage users in empathetic conversations, providing guidance, coping strategies, and referrals to professional assistance and other available support services. By delivering personalized support and knowledge dissemination, chatbots effectively bridge the information gap that often surrounds mental health concerns, thereby encouraging individuals to seek help when warranted. Psychoeducation constitutes a pivotal component of mental health awareness endeavors, offering individuals essential knowledge and coping strategies. The AI-enhanced educational platforms further accentuate this by leveraging data mining techniques to curate tailored content for catering to the diverse needs of users. This personalization enhances user engagement, ensuring that educational material is both relevant and relatable to individual experiences, thereby fostering a deeper understanding of mental health concepts.
• Support: How AI can provide support to individuals with mental health concerns? Mental health concerns present a global challenge, necessitating innovative approaches to support individuals on their path to well-being. The convergence of AI and mental health care has spurred the adoption of unique solutions that complement traditional methods.

Mental healthcare professionals can actively employ various AI-driven client engagement strategies to effectively guide the recovery journey for individuals grappling with mental health issues. For instance, AI technologies can be seamlessly integrated into mobile applications to send timely reminders for medication schedules, track side effects, monitor medication responses, enhance adherence, and facilitate collaboration between individuals and their healthcare providers. AI-driven apps can also monitor mood fluctuations and offer insights into potential triggers, enabling individuals to identify patterns and make informed decisions about self-care.

Personal sensing, also known as digital phenotyping, entails utilizing digital information to assess and observe an individual's mental well-being (37). Artificial intelligence can analyze content shared on social media platforms, medical records, and other sources. Through this analysis, AI can identify significant shifts in behavior that it has learned to correlate with mental health conditions. For instance, if an individual who regularly wears a smartwatch to monitor physical activity undergoes a sudden transition from high activity levels to a predominantly sedentary state, AI technology may interpret this change as a potential indicator of depression. This interpretation aligns with the typical pattern of reduced energy levels and diminished motivation to engage in physical exercise that is often observed in cases of depression. Platforms like Facebook have also integrated AI-driven tools that identify concerning posts and offer assistance. Furthermore, AI can foster connections among individuals facing similar challenges by facilitating online support groups and communities, where individuals can exchange experiences and strategies. Additionally, AI-based applications, such as tracking an individual's progress over time and providing feedback on their efforts, can promote sustained motivation for enhanced recovery.

• Intervention: How AI can assist in the intervention and treatment of mental health disorders?

Modern AI and machine learning, in particular, present extensive possibilities for advancing prediction, detection, and treatment solutions in the domain of mental health care. This technology has the capacity to not only assist mental health practitioners in redefining mental illnesses more objectively than the current DSM-5 framework but also to identify these conditions at earlier, prodromal stages when interventions can yield maximum efficacy. Moreover, AI enables the personalization of treatments based on an individual's unique characteristics. In the context of prediction and prevention, AI can evaluate the risk of developing specific mental health disorders based on an individual's profile, genetic predisposition, and environmental factors. This enables proactive measures to prevent or mitigate the onset of disorders. An AI-based decision support system (DSS) has been developed, efficiently detecting and diagnosing various mental disorders (38). AI algorithms can sift through diverse data sources, such as electronic health records, diagnostic tests, and behavioral patterns, for early detection of mental health disorder signs, allowing timely intervention, and improved prognosis.

In terms of treatment, numerous studies have indicated that NLP-based chatbots possess the capability to identify mental health issues through a question-based approach similar to that of mental health practitioners (39). For instance, chatbots may inquire about various aspects, including mood, stress levels, energy, and sleep patterns (40). Subsequently, these responses are analyzed, enabling the chatbot to recommend various therapeutic techniques. These recommendations include purely behavioral modifications, such as engaging in activities like walking, meditation, and relaxation techniques, or guiding the individual to seek medical support. Furthermore, in instances where the patient's immediate safety is a concern, the chatbot could promptly notify their healthcare provider (37). Expanding on therapeutic interventions, AI can augment traditional therapy approaches by delivering cognitive behavioral exercises and interventions through digital platforms. These tools reinforce learning, provide consistent support, and track progress over time. AI-powered mental health applications provide accessible and convenient support to individuals, particularly those who may have limited access to traditional therapy services, offering on-demand assistance and interventions, and further reducing barriers to seeking help. Additionally, AI-driven neurofeedback systems and brain-computer interfaces offer novel ways of regulating brain activity and emotional states. These interfaces provide immediate feedback on mental states and enable individuals to develop self-regulation skills. Furthermore, AI can play a crucial role in the analysis of aggregated patient data, generating insights and recommendations for clinicians to make sound treatment decisions, thereby optimizing clinical outcomes.

Beyond the broad spectrum of AI tools enhancing awareness, support systems, and interventions in mental health, it's equally imperative to delve into the specific advantages these AI-driven applications bring to the table.

## Advantages of AI applications in mental health outcomes

### Positive impact on cognitive aspects

The inaccuracy and subjectivity of cognitive assessments have led many healthcare professionals to explore tools and techniques that would automate these processes, making them more objective and increasing the efficiency of facilitation. AI has emerged as one of the most promising approaches to automate cognitive assessments. Cognitive assessments involve a series of tasks and tests that are typically designed to evaluate areas of cognitive function like language, reasoning, memory, decision making, attention and perception. These assessments are usually administered by healthcare professions like occupational therapists, neurologists or psychologists.

Screening of cognitive deficits or impairments and early intervention is currently the most widely accepted strategy to manage a number of psychological disorders. The diagnosis of these is established through thorough assessments, which may also help in understanding cognitive pathophysiology (41). However, lack of proper standardized screening and guidelines often leads to undiagnosed cognitive impairment which further leads to increased disease progression and cognitive decline.

Automating the assessment and prediction process is the key to timely diagnosis and management. The advent of AI has resulted in automated assessment techniques which improve the accuracy of diagnosis. ML and AI-based approaches like Support Vector Machine (SVM), neural networks and ensemble techniques like Convolutional Neural Network (CNN), AlexNet, GoogLeNet and LeNet5 have yielded some of the best results and accuracies when it comes to the use of AI for the assessment of cognitive mental health disorders (42).

The evolution of AI has contributed effectively to the early detection, diagnosis, and referral management of mental health disorders because experts are limited in regard to their performance, knowledge diversity and daily exertion which can also affect their performance.

#### Intellectual and developmental disorders

These include disorders like cerebral palsy, down syndrome, ADHD, autism spectrum disorders and fragile X syndrome which typically appear in a child before the age of 18. Data from different sources form inputs for the analysis of intellectual and developmental disabilities. The neuroimaging data are analyzed with a DNN to detect the presence of ID or DD in children. Based on this, AI-assisted screening systems have been developed to analyze the electronic health record of individuals for the detection of various disabilities and disorders. Machine learning can be used to detect the presence of disorders like ASD using eye movements. In addition, AI finds its role in the detection of ASDs from the presence of maternal and blood autoantibody-based biomarkers (43).

#### Neurodegenerative disorders

Major neurodegenerative disorders like Alzheimer's, Parkinsons and motor neuron disease possess a great challenge in that their symptoms are not seen until a substantial number of neurons are lost (44). Against this, early diagnosis is difficult. Using machine learning algorithms to analyze MRI images, early detection of these diseases is made easier. A study by Kloppel et al. (45) showed that Support Vector Machine (SVM) can use MRI scans to efficiently differentiate between individuals with Alzheimer's and those with frontotemporal lobar degeneration. Furthermore, they also helped distinguish between healthy individuals and those with Alzheimer's. 3D neural network architectures have also been used for the detection of Alzheimer's in the past.

#### Seizures

Epileptic seizures typically develop with a sudden abnormal surge of electrical activities in the brain. The detection of these seizures can prove to be a real challenge due to the variability in their pattern. Previous studies have demonstrated how electroencephalography recordings have been analyzed using machine learning algorithms for the effective detection of seizures (46).

### Positive impact on affective/emotional aspects

The integration of AI and affective computing has given rise to a realm known as Emotional AI, wherein technologies are designed to perceive, learn from, and interact with human emotions. Despite being in its nascent stages, Emotional AI is gaining prominence in various facets of daily life, from personal devices to professional domains. It is even influencing the emotional ambiance of spaces like workplaces, hospitals, and classrooms. Emotion sensing, a pivotal aspect of Emotional AI, traces its origins to affective computing in the 1990s (47). Enabled by weak, narrow, and task-based AI, Emotional AI aims to comprehend and interact with emotional states by analyzing a spectrum of data related to words, images, facial expressions, gaze direction, gestures, voices, and physiological signals, such as heart rate, body temperature, respiration, and skin conductivity (48). The input features for emotion recognition could include facial expressions, voice samples, or biofeedback data, while the output encompasses emotional states used for various purposes. Common machine learning techniques like convolutional neural networks, region proposal networks, and recurrent neural networks are frequently employed for these tasks. These emotional states are then utilized to enhance interactions with devices and media content, intensify artistic expression, facilitate surveillance and learning, and enhance self-understanding of moods and well-being (49).

This technology transcends human limitations, enabling more nuanced and accurate detection of emotional signals through wearables and smartphone applications. Furthermore, AI facilitates emotion regulation through tailored interventions. On the basis of real-time emotion data, AI-driven cognitive computing systems can offer immediate strategies, interactive exercises, and simulations for managing emotional states. For instance, AI-powered applications can guide users through relaxation exercises, deep breathing techniques, or mindfulness practices for reducing perceived stress and improving self-regulation (50).

The role of AI in emotional well-being extends beyond mere detection and regulation. AI-powered systems can also contribute to the development of emotional intelligence, a key factor in maintaining balanced emotional states. By providing users with insights into their emotional patterns over time, AI empowers individuals to cultivate a deeper understanding of their emotions and triggers, equipping individuals with valuable skills for effectively managing emotions and fostering healthier responses to challenges. Furthermore, AI-driven interfaces and applications can adapt their responses based on users' emotional cues, providing empathetic and supportive interactions. These emotionally intelligent interfaces offer a human-like connection, enhancing users' emotional experiences and addressing their emotional needs.

#### Emotional dysregulation

Difficulties in effectively managing and expressing emotions, often lead to heightened emotional responses, mood instability, and impaired emotional functioning, increasing the risk of depression, anxiety, and substance use disorders. Music-based emotion regulation mobile app has been developed with the aim of teaching emotion regulation skills to individuals with mental health problems in clinical and community settings, including eating disorders, anxiety disorders, substance misuse, and schizophrenia (51). AI-powered therapeutic games and virtual reality experiences can also provide immersive environments for practicing emotion regulation skills. These interactive platforms offer safe spaces to explore and manage emotions, allowing individuals to gradually build their emotional regulation capacities. Furthermore, AI-enabled biofeedback and neurofeedback systems can help individuals gain awareness and control over their physiological responses associated with emotional dysregulation. These systems provide real-time feedback on heart rate, brain activity, and other physiological indicators, allowing individuals to learn how to modulate their emotional responses. Additionally, immediate and empathetic support can be offered by AI-driven virtual therapists or chatbots for individuals experiencing emotional dysregulation.

#### Mood disorders

The use of AI techniques holds significant potential in improving the diagnosis of mood disorders as well as identifying suicide risks (52). A combination of using mobile and wearable technology can assist in collecting physiological and behavioral markers followed by the AI to analyze these data that can provide objective markers for conditions such as depression and bipolar disorder (BD) (53). AI also has the capacity to monitor social media and text messages for linguistic cues and sentiment analysis, enabling the prediction of mood fluctuations and potential relapses. For example, the Social Rhythm Metric (SRM), a clinically validated marker of stability and rhythmicity for individuals with bipolar disorder (BD), can be automatically assessed using passively sensed data from smartphones (54). This personalized monitoring enhances self-management and reduces the risk of severe episodes.

#### Autism spectrum disorder (ASD)

AI-driven tools assist in early detection by analyzing facial expressions, eye gaze, and gestures during video-based interactions. Smart tablet technology can provide a new paradigm for clinical autism assessments, aiding in the identification and intervention of ASD in young children (55). The capability of this technology extends to its integration with customized gaming experiences that incorporate various psychometric assessments. Additionally, it has the capacity to utilize sensors in innovative manners, including evaluating social intelligence or detecting emotional responses through the front-facing camera or by combining gameplay with sensor-equipped toys for enhanced functionality (56).

#### Schizophrenia

Automated speech analysis can assess speech patterns and prosody to detect subtle and clinically relevant affect-related changes in speech that might indicate the onset of schizophrenia symptoms (57). NLP-driven tools can also assist in tracking and predicting relapses based on speech patterns and emotional content (58). AI interventions can play a crucial role in promoting emotional well-being among children and adolescents. Interactive and engaging AI-driven platforms can provide age-appropriate tools for emotional regulation and stress management. These platforms may include virtual reality experiences, interactive games, and personalized virtual companions. AI can also monitor online activities and social media usage to detect signs of cyberbullying, anxiety, or depression in young users. Early intervention through AI can help develop healthy emotional coping mechanisms and prevent the escalation of mental health issues.

For young adults transitioning into adulthood, AI applications can offer support in managing the stresses of higher education, career choices, and independent living. AI-powered virtual mentors can provide guidance on stress reduction techniques, time management, and decision-making. Additionally, AI-driven platforms can curate resources for building resilience, coping with life changes, and maintaining a healthy work-life balance. Middle-aged adults often face increased responsibilities and societal pressures. AI can assist this group by offering personalized stress management strategies, facilitating relaxation techniques, and providing reminders for self-care activities. AI-powered chatbots or virtual therapists can offer a confidential space for discussing emotional concerns, and AI-based wellness platforms can tailor fitness and wellness routines to individual preferences and schedules.

The emotional challenges in the elderly can be effectively addressed by AI applications, including feelings of isolation, cognitive decline, and age-related mental health issues. AI-driven virtual companions can offer companionship and engage older adults in cognitive exercises and reminiscence therapy. Additionally, AI-powered sensors can detect changes in behavior patterns and alert caregivers or healthcare providers to potential emotional distress or cognitive decline.

While acknowledging the substantial strides made by AI in enhancing mental health care, it is important to scrutinize these advancements and address the nuanced concerns surrounding their implementation. Bridging the gap between the perceived benefits and potential drawbacks of AI applications in mental health outcomes requires a critical evaluation.

## Critiques of AI applications in mental health outcomes: bridging the Gap

1. Ethics and privacy concerns related to AI use in mental health care

The use of artificial intelligence (AI) in mental healthcare has the potential to offer numerous benefits, such as improved diagnostics, personalized treatment, and increased access to mental health support. However, it also raises important ethical and privacy concerns that must be carefully addressed to ensure responsible and effective implementation. Some of the issues of concern include – (59)

• Data Privacy and Security: AI systems in mental healthcare often require access to sensitive and personal patient data, including medical records, treatment histories, and even real-time emotional states. Safeguarding this data is crucial to protect patient privacy and prevent unauthorized access or breaches (60).
• Informed Consent: Patients should be fully informed about how their data will be used and the potential implications of AI-driven mental health interventions. Informed consent is especially important considering the sensitive nature of mental health information (61).
• Transparency: AI algorithms used in mental healthcare should be transparent and explainable. Patients and healthcare providers need to understand how decisions are made by AI systems to ensure accountability and to build trust.
• Bias and Fairness: AI algorithms can inherit biases present in the data they are trained on, leading to potential disparities in diagnosis and treatment recommendations. Efforts must be made to identify and mitigate these biases to ensure fair and equitable care for all individuals.
• Human Oversight: While AI can assist mental healthcare professionals, it should not replace human expertise entirely. Maintaining a balance between AI-driven recommendations and human judgment is crucial to avoid potential errors and to uphold the ethical responsibility of healthcare providers.
• Accountability and Liability: When AI systems are involved in making decisions about mental healthcare treatment, questions about accountability and liability arise. Determining responsibility in case of adverse outcomes caused by AI recommendations can be complex and needs careful consideration.
• Patient-Provider Relationships: The use of AI could potentially alter the dynamics of the patient-provider relationship. Maintaining empathy, trust, and human connection in mental healthcare interactions is essential, even when AI tools are used.
• Unintended Consequences: AI systems might inadvertently reinforce stigmatization, overdiagnosis, or unnecessary medicalization of normal emotional experiences. Careful monitoring and adjustment of AI algorithms are needed to avoid such unintended consequences.
• Regulation and Standards: Clear regulatory frameworks and ethical guidelines should be established to govern the development, deployment, and use of AI in mental healthcare. These standards should ensure that patient rights, privacy, and well-being are protected.

Collaboration between AI developers, mental healthcare professionals, ethicists, and policymakers is essential to create responsible and effective AI solutions that prioritize patient well-being and uphold ethical standards.

2. Reliability and accuracy issues of AI algorithms in assessing and diagnosing mental health disorders

While AI has shown promise in assisting clinicians and providing insights, there are several considerations to take into account like the quality of data on which the algorithm is based. The accuracy of AI algorithms heavily depends on the quality and diversity of the data they are trained on. If the training data is biased, incomplete, or unrepresentative, the AI may produce inaccurate or biased results. Mental health disorders are complex and often involve a combination of subjective symptoms, environmental factors, and personal histories. AI algorithms may struggle to accurately diagnose conditions that require nuanced interpretation of these factors. The human aspects and reading in between the lines of a patient's history may be missed by algorithms. Mental health disorders can manifest differently in different individuals (62). This variability challenges the development of a one-size-fits-all AI solution and requires algorithms that can adapt to diverse presentations. AI algorithms can produce false positives (diagnosing a disorder that isn't present) and false negatives (failing to diagnose a disorder that is present). AI algorithms may lack the ability to fully understand the context and nuances of a patient's life, emotions, and experiences, which can affect accurate diagnosis and assessment. Many mental health disorders have overlapping symptoms, making accurate diagnosis challenging even for experienced clinicians. AI algorithms may struggle with this complexity as well (63).

The field of mental health is continuously evolving, with updates to diagnostic criteria and new insights. AI algorithms need to be regularly updated to stay aligned with the latest research. AI algorithms should be seen as tools to assist mental health professionals rather than replace them. Human oversight is crucial to review and interpret AI-generated assessments. AI algorithms for mental health diagnosis should undergo rigorous validation and testing similar to any diagnostic tool. Clinical trials are necessary to demonstrate their reliability and safety.

3. Potential biases and lack of cultural sensitivity in AI-based interventions

This is another significant concern when it comes to AI-based interventions in mental health. AI algorithms learn from historical data, which may reflect societal biases and inequalities. If the training data is biased, the AI system can perpetuate those biases in its decision-making. This can lead to disparities in diagnosis, treatment recommendations, and outcomes for different demographic groups. Mental health is influenced by cultural, social, and contextual factors. AI tools may not adequately account for these factors, leading to misinterpretations or misunderstandings of patients' experiences and needs. A lack of cultural sensitivity can result in inaccurate diagnoses and recommendations. If certain demographic groups are underrepresented in the training data, AI algorithms may not be as accurate for those groups. This is particularly concerning for minority populations, as the algorithms may not effectively capture the nuances of their mental health experiences. Biased algorithms can wrongly label individuals or stigmatize certain communities (64). For example, misdiagnosing cultural behavior as a mental disorder can perpetuate negative stereotypes. AI algorithms might misinterpret cultural expressions of distress or emotional states, leading to misdiagnosis or ineffective interventions. Different cultures may have unique ways of expressing and coping with mental health challenges. AI tools might not understand or communicate effectively in various languages and dialects, limiting their accessibility to diverse populations. Different cultures have diverse beliefs about mental health, help-seeking, and treatment. AI tools need to respect and adapt to these cultural norms to provide relevant and effective interventions. Creating AI solutions with cultural sensitivity requires diverse development teams that reflect a variety of backgrounds and perspectives. This helps in addressing biases and designing interventions that are relevant across cultures. Collaboration between AI developers and mental health professionals with cultural expertise is crucial. This collaboration ensures that AI tools are well-informed about cultural nuances and potential biases (65).

Understanding the significance of refining AI's role and addressing the critical aspects of implementation in mental health care, the following section delineates key recommendations aimed at fortifying these technologies.

### Recommendations for improving AI applications in mental health care

1. Enhanced training and validation of AI algorithms with diverse datasets

One must collect a wide range of data that represent different demographic groups, cultural backgrounds, languages, and socioeconomic statuses. Ensure that the dataset is balanced and includes sufficient samples from underrepresented populations. One must obtain explicit informed consent from individuals contributing data. Anonymize and protect sensitive information to maintain privacy and confidentiality. The AI developer must conduct thorough bias assessments to identify potential biases in the dataset. One must use techniques such as debiasing algorithms to reduce biases in training data (66).

One must consider the intersectionality of various demographic factors (e.g., race, gender, age) to ensure that the dataset captures the complexities of individuals' identities. There is a need to collaborate with mental health professionals, cultural experts, and ethicists to guide the selection and curation of diverse datasets and to involve domain experts in the development of bias-mitigation strategies. There is a need to develop AI models that can adapt to different cultural contexts and languages. One must ensure that data labeling takes into account diverse perspectives and cultural norms. There is a need to establish clear guidelines for labeling subjective data related to mental health symptoms. Research must validate the AI algorithm's performance across different demographic groups separately to identify potential disparities. The developers must regularly audit AI models for biases and accuracy and update them as new data becomes available and stay current with evolving research and best practices in AI fairness and ethics (67).

2. Implementation of transparent and accountable AI systems

There is a need to develop AI algorithms that provide explanations for their decisions and recommendations in a human-readable manner and use techniques like interpretability tools and visualizations to make AI processes understandable to non-experts. One must maintain detailed documentation that describes the AI system's architecture, data sources, training process, algorithms used, and decision-making logic while ensuring that the documentation is easily accessible to stakeholders, including developers, regulators, and users. The developers could consider open-sourcing parts of the AI system's code to allow external experts to review and audit the technology for biases, vulnerabilities, and ethical concerns. There is a need to conduct thorough assessments of the potential impact of AI algorithms on various stakeholders, including marginalized communities, and take steps to mitigate any negative effects. One could also educate users about how AI systems work, their limitations, and the extent to which human oversight is involved and provide resources and materials to help users make informed decisions about using AI technologies. One must regularly audit AI systems for biases and fairness issues, especially related to factors like race, gender, and socioeconomic status while addressing and rectifying any identified biases to ensure equitable outcomes. There is a need to establish ethics committees or review boards that include experts from various fields, including ethics, law, and social sciences, to assess and oversee AI system deployment. One could collaborate with third-party organizations or auditors to conduct independent assessments of the AI system's transparency, accountability, and ethical practices (68, 69).

3. Integration of human oversight and collaboration in AI-based mental health interventions

It is vital that we position AI as a tool to assist mental health professionals rather than replace them and emphasize the complementary roles of AI and human expertise. There is a need to foster a collaborative approach where AI provides insights and recommendations, while human professionals make the final decisions. One must involve mental health experts, psychologists, therapists, and counselors in the design and development of AI interventions. Their domain knowledge is crucial for creating effective tools. There is a need to collaborate with mental health professionals to validate the accuracy and clinical relevance of AI algorithms and use clinical trials and real-world testing to ensure that AI interventions align with best practices in mental healthcare. One must allow mental health professionals to customize AI algorithms to fit individual patient needs and preferences and leverage human insights to fine-tune AI recommendations for each patient. One must develop AI systems that provide understandable explanations for their recommendations.

Mental health professionals can better trust and act upon AI recommendations when they understand the reasoning behind them. One must encourage mental health professionals to regularly review and monitor AI-generated insights and create channels for professionals to provide feedback and corrections to improve the accuracy and relevance of AI suggestions. There is a need to collaborate with culturally competent mental health experts to ensure that AI interventions are sensitive to diverse cultural backgrounds and practices. AI must reserve complex and high-risk cases for human intervention. AI can be effective for routine tasks, leaving more nuanced cases to human experts (70).

One must strive to maintain a strong patient-provider relationship by incorporating human empathy and emotional support. One must use AI as a supplement to enhance patient care, not a replacement for human connection and provide mental health professionals with ongoing training on how to effectively use AI tools and interpret AI-generated insights. By integrating human oversight and collaboration, AI-based mental health interventions can benefit from both technological advancements and the nuanced understanding that only human professionals can provide. This approach helps to strike a balance between innovation and the ethical delivery of quality mental healthcare (65).

## Conclusion

AI has come a long way from the initial Turing test to its current applications and is increasingly being integrated into digital medicine to contribute to mental health research and practice. The synergy between these domains holds immense potential for advancing our understanding, diagnosis, and treatment of mental health disorders. The insights of this study, however, are not without limitations. The primary constraints lie in the generalizability of the findings, as the scope of the review may not encapsulate the entirety of AI's impact in mental health.

Furthermore, the fast-paced nature of technological advancements implies that the landscape of AI applications in mental health is continually evolving, warranting ongoing research and adaptation.

Despite these limitations, this review underscores the complementary relationship between AI and healthcare by describing how the two fields help each other to promote positive mental health and places emphasis on the pivotal role of collaborative efforts between diverse experts in driving the responsible integration of AI into mental healthcare.

A study by De Choudhury et al. (71), illustrated that an important step is combining human intelligence with AI in order to (1) ensure construct validity, (2) appreciate unobserved factors not accounted for in data, (3) assess the impact of data biases, and (4) proactively identify and mitigate potential AI mistakes. In order to realize and tap into the full potential of AI, a wide community of experts in mental healthcare and research like clinicians, scientists, patients and regulators must collaborate and communicate effectively.

In this evolving landscape, it is also crucial to balance the immense potential of artificial intelligence with ethical responsibilities. We must consistently assess and refine its use to ensure it aids human abilities rather than replacing them. Continuous ethical scrutiny is vital to keep AI as a tool that supports us rather than overtaking our roles.

The future of AI in mental healthcare is indeed promising. Researchers and practitioners that are vested in improving mental healthcare must take an active role in informing the introduction of AI into clinical care by lending their clinical expertise and collaborating with data and computational scientists, as well as other experts, to help transform mental health practice and improve overall care for patients.
